# “Darwin’s butterflies”? DNA barcoding and the radiation of the endemic Caribbean butterfly genus *Calisto* (Lepidoptera, Nymphalidae, Satyrinae)

**DOI:** 10.3897/CompCytogen.v5i3.1730

**Published:** 2011-08-24

**Authors:** Andrei Sourakov, Evgeny V. Zakharov

**Affiliations:** 1 McGuire Center for Lepidoptera and Biodiversity, Florida Museum of Natural History, University of Florida, Gainesville, FL 32611, USA; 2 Biodiversity Institute of Ontario, University of Guelph, Guelph, ON, Canada N1G 2W1

**Keywords:** COI, biogeography, DNA barcoding, islands, intraspecific variation, Lepidoptera, Nymphalidae, Satyrinae, speciation, taxonomy

## Abstract

The genus *Calisto* Hübner, 1823 is the only member of the diverse, global subfamily Satyrinae found in the West Indies, and by far the richest endemic Caribbean butterfly radiation. *Calisto* species occupy an extremely diverse array of habitats, suggestive of adaptive radiation on the scale of other classic examples such as the Galápagos or Darwin’s finches. However, a reliable species classification is a key requisite before further evolutionary or ecological research. An analysis of 111 DNA ‘barcodes’ (655 bp of the mitochondrial gene COI) from 29 putative *Calisto* species represented by 31 putative taxa was therefore conducted to elucidate taxonomic relationships among these often highly cryptic and confusing taxa. The sympatric, morphologically and ecologically similar taxa *Calisto confusa* Lathy, 1899 and *Calisto confusa debarriera* Clench, 1943 proved to be extremely divergent, and we therefore recognize *Calisto debarriera* **stat. n.** as a distinct species, with *Calisto neiba* Schwartz & Gali, 1984 as a junior synonym **syn. n.** Species status of certain allopatric, morphologically similar sister species has been confirmed: *Calisto hysius* (Godart, 1824) (including its subspecies *Calisto hysius aleucosticha* Correa et Schwartz, 1986, **stat. n.**), and its former subspecies *Calisto batesi* Michener, 1943 showed a high degree of divergence (above 6%) and should be considered separate species. *Calisto lyceius* Bates, 1935/*Calisto crypta* Gali, 1985/*Calisto franciscoi* Gali, 1985 complex, also showed a high degree of divergence (above 6%), confirming the species status of these taxa. In contrast, our data suggest that the *Calisto grannus* Bates, 1939 species complex (including *Calisto grannus dilemma* González, 1987, *Calisto grannus amazona* González, 1987, **stat. n.**, *Calisto grannus micrommata* Schwartz & Gali, 1984, **stat. n.**, *Calisto grannus dystacta* González, 1987, **stat. n.**, *Calisto grannus phoinix* González, 1987, **stat. n.**, *Calisto grannus sommeri* Schwartz & Gali, 1984, **stat. n.**, and *Calisto grannus micheneri* Clench, 1944, **stat. n.**) should be treated as a single polytypic species, as genetic divergence among sampled populations representing these taxa is low (and stable morphological apomorphies are absent). A widely-distributed pest of sugar cane, *Calisto pulchella* Lathy, 1899 showed higher diversification among isolated populations (3.5%) than expected, hence supporting former separation of this species into two taxa (*pulchella* and *darlingtoni* Clench, 1943), of which the latter might prove to be a separate species rather than subspecies. The taxonomic revisions presented here result in *Calisto* now containing 34 species and 17 subspecies. Three species endemic to islands other than Hispaniola appear to be derived lineages of various Hispaniolan clades, indicating ancient dispersal events from Hispaniola to Puerto Rico, Cuba, and Jamaica. Overall, the degree of intrageneric and intraspecific divergence within *Calisto* suggests a long and continuous diversification period of 4–8 Myr. The maximum divergence within the genus (ca. 13.3%) is almost equivalent to the maximum divergence of *Calisto* from the distant pronophiline relative *Auca* Hayward, 1953 from the southern Andes (14.1%) and from the presumed closest relative *Eretris* Thieme, 1905 (14.4%), suggesting that the genus began to diversify soon after its split from its continental sister taxon. In general, this ‘barcode’ divergence corresponds to the high degree of morphological and ecological variation found among major lineages within the genus.

## Introduction

The genus *Calisto* Hübner, 1823 is endemic to the West Indies, and, until the present revision, comprised 54 named taxa (Lamas et al. 2004) of small to medium sized butterflies in the subfamily Satyrinae, a diverse global radiation including ca. 2,200 described species. *Calisto* is considered a member of the neotropical subtribe Pronophilina, but while many *Calisto* are lowland dwellers, occurring as low as sea level, almost all other pronophilines are exclusively montane and/or temperate. Most of the extant described species of *Calisto* are found on the island of Hispaniola, with a single species on Jamaica, one on Puerto Rico, two species on the Bahama Islands, one on Anegada, and two on Cuba (Smith et al. 1994).

Though *Calisto* are neither visually spectacular nor economically important (with the exception of *Calisto pulchella* Lathy, 1899 , which is a pest of sugar cane), a significant amount of information is available on the distribution of the more common species on Hispaniola from the general survey of the island’s butterflies by Schwartz (1989). However, phylogenetic relationships of the genus are unclear and affinities to both South American and African taxa have been proposed based on adult morphology (Riley 1975, Miller and Miller 1989), although the most recent taxonomic treatments of the tribe (e. g., Viloria 1998; Lamas et al. 2004) kept *Calisto* in Pronophilina. The montane neotropical genus *Eretris* Thieme, 1905 has been considered one of the closest relatives of *Calisto* by some (Miller 1968, De Vries 1987, Peña et al. 2011).

The morphology of immature stages has been utilized extensively in phylogenetic studies of butterflies (e. g., Kitching 1985, Murray 2001, Penz and DeVries 2001, Freitas and Brown 2004, Willmott and Freitas 2006), however this has been mostly at higher taxonomic levels. *Calisto* is one of the few satyrine genera for which the immature stages have been studied in detail at least for most major species groups, providing insights into a high degree of morphological diversification in the egg and larvae, atypical for other satyrine genera (Sourakov 1996, 2000). Structures that normally show little variation in the Satyrinae intragenerically, such as male and female genitalia, are also remarkably diverse in *Calisto* (Sourakov 1997). Until now, however, it has been unclear whether this spectacular morphological variation results from an ancient history of divergence, or from strong disruptive selection on traits potentially involved with fitness and reproductive isolation.

Many species of *Calisto* were described only recently, towards the end of the 20th century (e.g., Schwartz and Gali 1984, Gali 1985, Johnson and Hedges 1998) and are still known only from the type series. Small numbers of specimens, in conjunction with reliance on wing pattern elements alone, which often seem to be variable in better known taxa, makes the status of many of these recent names difficult to determine. For example, additional eyespots were used to define the species *Calisto neiba* Schwartz & Gali, 1984 and *Calisto amazona* González, 1987. Many of these names might thus prove to be synonyms, or, conversely, represent a formerly unexplored array of cryptic species that are only just being recognized. The taxonomic confusion is evident in Smith et al.’s (1994) comprehensive treatment of Caribbean butterflies; they listed all the described taxa, but for many species avoided illustrating them and provided inconclusive comments on the validity of many taxa. For instance, they did not illustrate *Calisto montana* Clench, 1943 for which only the male holotype is known, and of which even the precise collecting locality is uncertain. For *Calisto neiba*, Smith et al. (1994) stated that it has additional wing ocelli (which are, however, a variable character in many Satyrinae (e. g., Sourakov 1995, Kooi et al. 1996), repeatedly appearing within all species of *Calisto*, usually as an occasional aberration), and concluded that “the final estimate of the affinities of *Calisto neiba* cannot yet be made.” Another un-illustrated species, *Calisto aleucosticha* Correa et Schwartz, 1986, described from a couple of individuals that could represent aberrant *Calisto batesi* Michener, 1943 females, was assessed as “very close to *Calisto hysius* (Godart, 1824), and discovery of the male may well clarify its status.” The illustration of *Calisto micheneri* Clench, 1944 represents a taxon similar to our concept of *Calisto grannus dilemma* González, 1987, a taxon not illustrated by Smith et al. (1994) and said to be known “from a single specimen only. It is readily confused with other common species, and may well be more frequent than the rather sparse records would suggest.” Also not illustrated were *Calisto phoinix* González, 1987, of which Smith et al. (1994) said that “there seems little doubt that this species is not conspecific with *Calisto grannus*, but their relationships remains to be established,” and *Calisto dystacta* González, 1987, which “occurs at lower altitude than *Calisto phoinix*. The two are very similar and may be conspecific.” We examined type specimens and the original descriptions of Schwartz and Gali (1984) and González (1987) and could only conclude that these names most likely represent variants of *Calisto grannus* Bates, 1939 found at unusual elevations and slopes, and hence exhibiting slightly different phenotypes from typical specimens of the latter taxon.

A different issue is presented by the taxa that are clearly allopatric (and probably remained in isolation for a long time), but which are so morphologically similar that one must question the extent of diversification between them. For instance, Smith et al. (1994) treat *Calisto batesi* as a separate species, following treatment by Schwartz (1989), yet state that “this insect has generally been considered a subspecies of *Calisto hysius*.” Originally, *batesi* was described as a subspecies of *hysius* and Smith et al. (1994) chose to illustrate *Calisto batesi*, but did not illustrate *Calisto hysius*, because, we presume, the main difference between these taxa aside from their distribution is their size (*batesi* 13–15 mm; *hysius* 16.5–17.5mm), while the wing patterns are identical.

We find allopatric similar taxa within other major species groups, such as *Calisto chrysaoros* Bates, 1935 (names include *Calisto galii* Schwartz, 1983 and *galii choneupsilon* Schwartz, 1985) and *Calisto lyceius* Bates, 1935 (names include *Calisto crypta* Gali, 1985 and *Calisto franciscoi* Gali, 1985). In the *Calisto confusa* Lathy, 1899 complex, the name *Calisto confusa debarriera* Clench, 1943 has been attributed to a form with reduced white discal and extradiscal bands on the underside, which is found throughout the geographic range of *Calisto confusa confusa* and is occasionally sympatric, though frequently replaces typical *Calisto confusa* phenotypes at higher elevations. *Calisto montana* Clench, 1943 was described from the same group based on a single very worn specimen which had an unusual double-pupiled eye-spot on the underside of its forewing (Fig. 7) – a character found occasionally throughout *Calisto*. Other taxa within *Calisto confusa* species complex have also been described, such as *Calisto gonzalezi* Schwartz, 1988 for which Smith et al. (1994) state that “the exact relationship between this species and *Calisto confusa* remains to be clarified should new populations of *Calisto gonzalezi* be discovered.”

The above confusion over the recently described taxa is perhaps partly due to sole reliance of the authors on wing characteristics combined with distribution data in their approach to delineating new species, partly due to limited series and quality of specimens, and partly due to the exercising of the typological approach in its extreme form, with a disregard for interspecific variation. A possible solution to the problem is to use a new set of characters such as molecular sequence data. The technique of ‘DNA barcoding’ is based on the analysis of short, standardized gene regions; in the case of animals, this is a 655-bp segment of mitochondrial cytochrome oxidase subunit I (COI). DNA barcoding potentially provides an efficient method for species identification as well as for solving species-level taxonomical problems. Although the DNA barcode region can vary intraspecifically on a geographic scale as well as within populations (e. g., Lukhtanov et al. 2009, DeWalt 2011), and has shown varying degrees of success in species delimitation (e.g., Wiemers and Fiedler 2007), it has overall proved to be an excellent tool for species identification as illustrated in several large Holarctic Lepidoptera groups (Hebert et al. 2010, Lukhtanov et al. 2009). In the present study, therefore, we explore the potential for DNA barcode data to attempt to answer long-standing questions concerning interspecific and intraspecific relationships within the genus by studying 21 species of *Calisto* (representing almost all of the major species groups). We examine a number of questionable taxa, such as representatives of *Calisto grannus* and *Calisto confusa*, and the *Calisto lyceius* species complex. Furthermore, this study allows us to examine the utility of the DNA barcoding method for species delimitation using a group, which, unlike the Holarctic fauna, probably underwent continuous diversification for a prolonged period without the major climatic stresses of glaciations. The results of this study should also add to our understanding of the extent to which DNA-barcode divergence correlates with morphological and ecological divergence. Prior to further phylogenetic work based on morphological, molecular or combined characters, it is key to establish species boundaries and the alpha taxonomy of the genus. In this study, we therefore use DNA barcodes to test the current species classification based on traditional characters, and to try to resolve the taxonomic status of a number of problematic phenotypes and populations.

## Methods

A total of 110 *Calisto* specimens representing 31 putative taxa were sampled (Table 1). All specimens were collected in 1994–1999 by the first author. None of the specimens were subjected to any chemical treatment before desiccation. The climate of the regions ensured quick drying of specimens, which were stored at a room temperature (18–25°C) for over 10 years. DNA was extracted from a single leg removed from each specimen. Specimens were mostly unprepared (papered), with the exception of several individuals.

We amplified a 655-bp segment of mitochondrial cytochrome oxidase subunit I, from the *COI* barcode region. All polymerase chain reactions and DNA sequencing were carried out following standard DNA barcoding procedures for Lepidoptera as described previously (Hajibabaei et al. 2006, deWaard et al. 2008). Photographs of all specimens used in the analysis as well as specimen collection data and sequences are available in the Barcode of Life Data System (BOLD) at http://www.barcodinglife.org/ as well as in GenBank (accession numbers JN197297--JN197406). All voucher specimens are deposited at the McGuire Center for Lepidoptera and Biodiversity (Florida Museum of Natural History, University of Florida).

We chose two genera as outgroups: *Eretris*, which Miller (1968) thought to be *Calisto*’s closest relative on the mainland, based on wing shape and relative proximity to the Caribbean, and the southern Andean genus *Auca* Hayward, 1953 (Satyrinae: Pronophilina), which we have observed to be morphologically and behaviorally similar to *Calisto* (e. g., *Auca*’s association with bunch grass in arid lowland habitats is very similar to species in the *Calisto lyceius* complex) (Sourakov pers. obs.). Though geographically distant from *Calisto*, the inclusion of such a Pronophilina member from the southern Andes could provide insight into the origin of *Calisto* should the genus prove to be non-monophyletic and also provides an additional point of comparison for the pairwise divergence analysis. Hence, we obtained five additional sequences from GenBank (table 1), including two species of *Auca*, *Auca coctei* (GenBank number DQ338833) and *Auca barrosi* (GenBank number DQ338832) (Peña et al. 2011), and two species of *Eretris*, *Eretris* sp. (GenBank number GQ357229) and *Eretris* sp.2 (GenBank number GQ864764) (Peña et al. 2006). We also obtained one additional sequence of *Calisto pulchella* (GenBank number GQ357225) (Peña et al. 2011).

Sequences were aligned using BioEdit software (Hall 1999) and manually edited. Sequence information was entered into the Barcode of Life Data System (http://www.barcodinglife.org) along with an image and collateral information for each voucher specimen. Detailed specimen records and sequence information, including trace files, are available in the LOWA project file in the BOLD website. All sequences are also available through GenBank.

Sequence data were analyzed using Bayesian inference (BI), as implemented in Mr Bayes 3.1.2 (Huelsenbeck and Ronquist 2001; Ronquist and Huelsenbeck 2003). A GTR substitution model with gamma-distributed rate variation across sites and a proportion of invariable sites was specified before running the program for 5,000,000 generations with default settings. The first 2500 trees (out of 10000) were discarded prior to computing a consensus phylogeny and posterior probabilities.

**Table 1.** *Calisto* species examined in the present study and resulting nomenclatural changes.

Smith & al. 1994 name	Describer(s)	Status change	Proposed new status
*Calisto aleucosticha*	Correa & Schwartz, 1986	stat. n.	*Calisto hysius aleucosticha*
*Calisto amazona*	González, 1987	stat. n.	*Calisto grannus amazona*
*Calisto arcas*	M. Bates, 1939		
*Calisto archebates*	(Ménétriés, 1832) (Satyrus)		
*Calisto batesi*	Michener, 1943		
*Calisto chrysaoros*	M. Bates, 1935		
*Calisto confusa*	Lathy, 1899		
*Calisto confusa debarriera*	Clench, 1943	stat. n.	*Calisto debarriera*
*Calisto crypta*	Gali, 1985		
*Calisto dystacta*	González, 1987	stat. n.	*Calisto grannus dystacta*
*Calisto eleleus*	M. Bates, 1935		
*Calisto franciscoi*	Gali, 1985		
*Calisto gonzalezi*	Schwartz, 1988	syn. n.	*Calisto debarriera*
*Calisto grannus*	M. Bates, 1939		
*Calisto grannus dilemma*	González, 1987		
*Calisto herophile*	Hübner, [1823]		
*Calisto hysius*	(Godart, [1824]) (Satyrus)		
*Calisto lyceius*	M. Bates, 1935		
*Calisto micheneri*	Clench, 1944, repl. name	stat. n.	*Calisto grannus micheneri*
*Calisto micrommata*	Schwartz & Gali, 1984	stat. n.	*Calisto grannus micrommata*
*Calisto montana*	Clench, 1943	syn. n.	*Calisto debarriera*
*Calisto neiba*	Schwartz & Gali, 1984	syn. n.	*Calisto debarriera*
*Calisto nubila*	Lathy, 1899		
*Calisto obscura*	Michener, 1943		
*Calisto phoinix*	González, 1987	stat. n.	*Calisto grannus phoinix*
*Calisto pulchella*	Lathy, 1899		
*Calisto pulchella darlingtoni*	Clench, 1943		
*Calisto raburni*	Gali, 1985		
*Calisto sommeri*	Schwartz & Gali, 1984	stat. n.	*Calisto grannus sommeri*
*Calisto tasajera*	González, Schwartz & Wetherbee, 1991		
*Calisto zangis*	(Fabricius, 1775) (Papilio)		

Maximum parsimony (MP) analysis was performed using a heuristic search as implemented in MEGA4 (Tamura et al. 2007). We used the close-neighbor-interchange algorithm with search level 3 (Nei and Kumar 2000) in which the initial trees were obtained by random addition of sequences (100 replicates). We used nonparametric bootstrap values (Felsenstein 1985) to estimate branch support on the recovered tree, with the bootstrap consensus tree inferred from 1000 replicates (MP tree is provided as Supplementary file). The Kimura 2-parameter model of base substitution was used to calculate genetic distances in MEGA4 software (Tamura et al. 2007). Dendroscope (Huson et al. 2007) was used to edit trees for publication.

## Results

**Figure 1.**
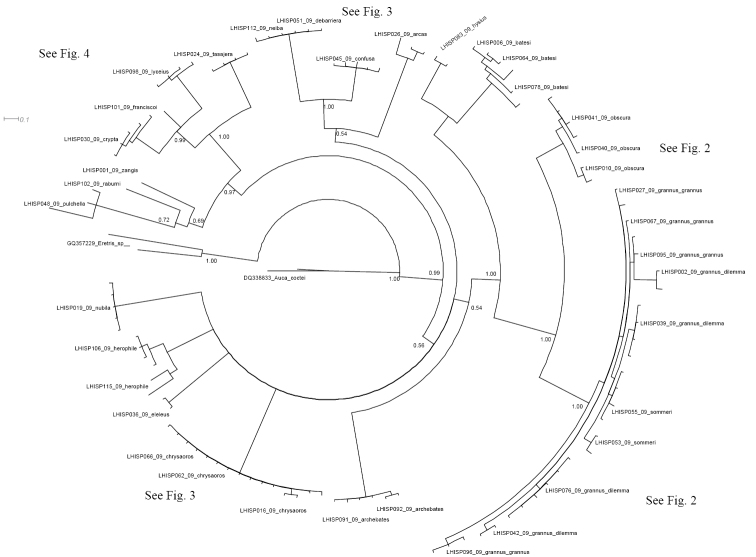
Bayesian inference phylogeny based on 655bp of COI for 111 specimens of the genus *Calisto* (representing ca. 20 species belonging to 26 named taxa), with outgroups of *Eretris* and *Auca* (Nymphalidae: Satyrinae: Prinophilini). The numbers at the nodes indicate posterior probability.

**Figure 2.**
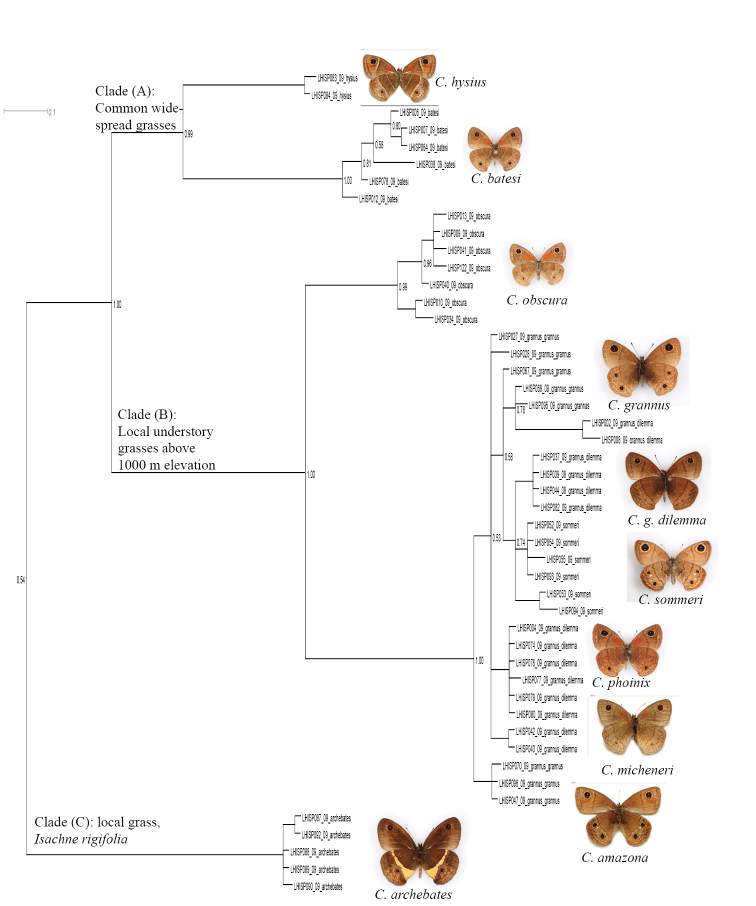
Fragment of the BI tree in Figure 1 with additional information about clades **Clade A:** *Calisto hysius* and *Calisto batesi* are found allopatrically on two Hispaniolan paleoislands**Clade B:** *Calisto obscura* is a widespread Hispaniolan species. The *Calisto grannus* complex is represented by a number of named populations, mostly but not exclusively found in Cordillera Central, the status of which are revised to subspecies in the present study **Clade C:** *Calisto archebates* is a local endemic of the southern paleoisland’s Sierra de Bahoruco.

Fig. 1 shows the results of the Bayesian Inference analysis (BI). The maximum parsimony analysis revealed a similar topology, but deeper nodes were not strongly supported (bootstrap value < 0.5). Bootstrap values higher than 0.5 are shown on the MP tree (see Supplementary file). For the further analysis and discussion of results we refer to the BI tree. The BI analysis of the tree topology and the Kimura 2-parameter model estimation of genetic distances showed the following results:

1. The sympatric, superficially similar widespread species *Calisto confusa*, *Calisto obscura* and *Calisto batesi*, which frequently share the same habitat, proved to be extremely divergent*. Calisto confusa* appear to be related to the morphologically highly derived *Calisto arcas* Bates, 1939 Fig. 3 (Clades A, B). *Calisto obscura*,which is found throughout the lowlands and mid-elevations proved to be related to the *Calisto grannus* species group which is found locally throughout the island at higher elevations (Fig. 2, Clade B). Though the latter clade has *Calisto batesi*/*Calisto hysius* species complex as its sister clade (Fig. 2, Clade A), the divergence between *Calisto obscura* and *Calisto batesi* is substantial at approximately 9%.

**Figure 3.**
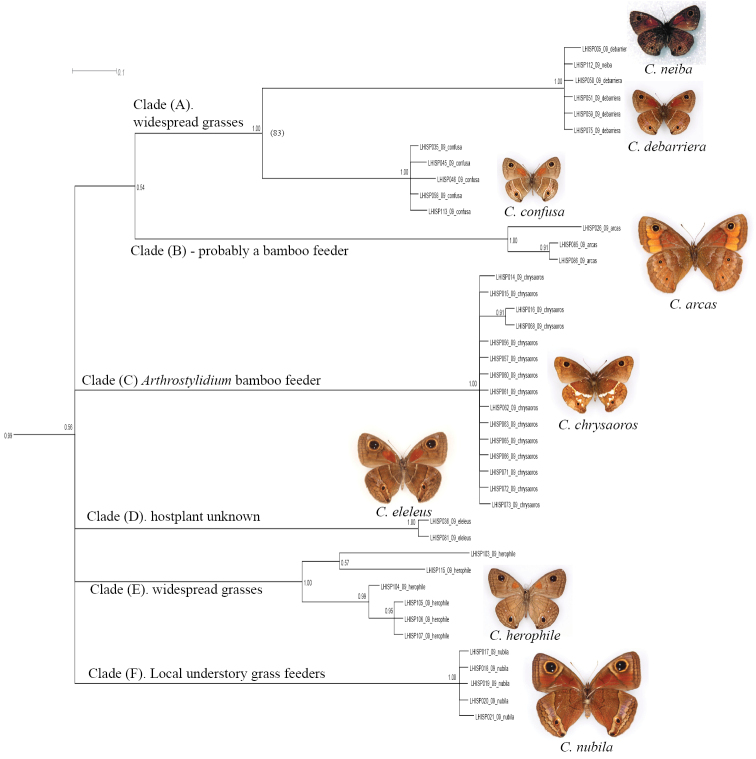
Fragment of the BI tree in Figure 1 with additional information about clades. **Clade A:** *Calisto confusa* and *Calisto debarriera*/*Calisto neiba* are morphologically similar and sometimes sympatric, though seemingly occupy different elevations**Clade B:** *Calisto arcas* is an endemic of Cordillera Central’s Valle Nuevo area**Clade C:** *Calisto chrysaoros* is found at high elevations on both southern and northern paleoislands in the refugias associated with climbing bamboo grass *Arthrostylidium* **Clade D:** *Calisto eleleus* is now found extremely locally in the Cordillera Central**Clade E:** *Calisto herophile* is distributed on Cuba and Bahamas islands **Clade F:** *Calisto nubila* is a Puerto Rican endemic.

**Figure 4.**
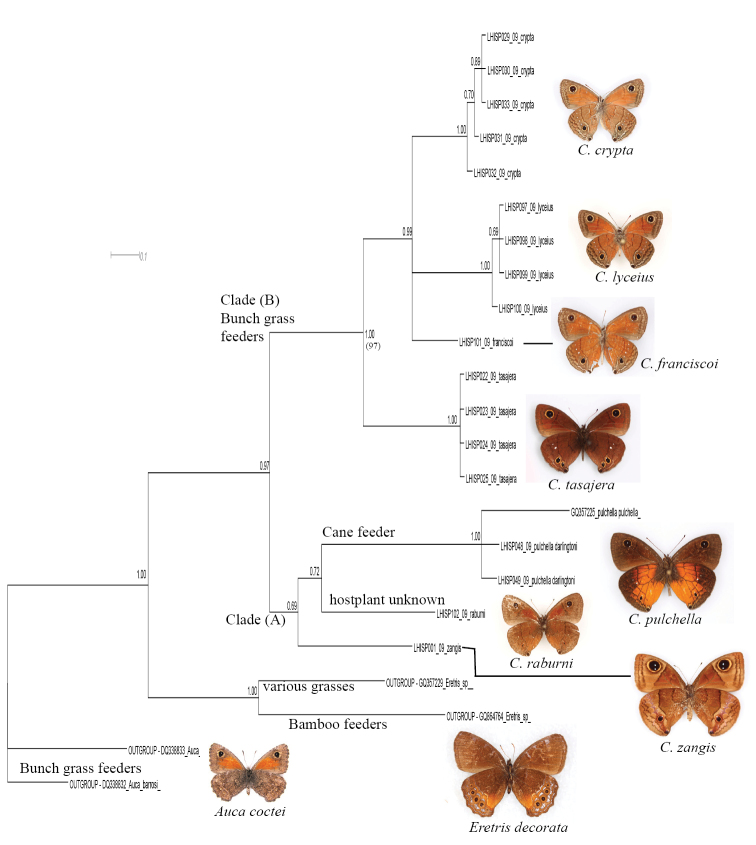
Fragment of the BI tree in Figure 1 with additional information about clades. The outgroups (*Auca* - bunch grass feeder from the southern Andes; *Eretris* - a bamboo-feeding group from Central and South America) and two basal *Calisto* clades **Clade A:** *Calisto zangis* of Jamaica which is aligned with the Hispaniolan *Calisto raburni* (a rare highly divergent species with an unknown life history) and *Calisto pulchella*, a well-known sugar cane pest (the native host plant is unknown) **Clade B:** *Calisto tasajera* (from the highlands of Cordillera Central) which feeds on *Danthonia domingenisis* bunch grass and *Calisto* of the *lyceius* group feeding on *Uniola virgata* bunch grass in the Hispaniolan lowlands.

2. The allopatric morphologically similar sister species *Calisto batesi*/*Calisto hysius* (Fig. 2, Clade A), whose species status was questionable based on adult morphology, and whose immature stages are also quite similar (Sourakov 1996), showed a high degree of divergence of ca. 6%, which is twice the rate seen in some sister species in Palearctic Satyrinae (Lukhtanov et al. 2009). For comparison, the divergence within *Calisto batesi* among well isolated populations throughout Cordillera Central, though still significant, is equal to or less than 1%.

3. *Calisto confusa* and *Calisto debarriera* appeared as two well-separated clusters (Fig. 3, Clade A)*. Calisto debarriera* was originally treated as subspecies of *Calisto confusa* (Munroe 1951), and later regarded as color variant of *Calisto confusa* because of its frequent sympatry with the latter (Sourakov per. obs.), and because rearing did not indicate additional morphological characters (Sourakov 1996, 1997). Individuals of both taxa used in our analysis came from the same localities throughout the island, and while they showed interspecific divergence of over 6%, showed divergence of less than ca. 0.2% intraspecifically. A single specimen with the phenotype of *Calisto neiba* (from Sierra de Neiba) was not divergent from the rest of *Calisto debarriera*, suggesting that the former is a synonym of the latter.

4. Within the *Calisto grannus* species complex (Fig. 2, clade B), we included at least nine isolated populations from different elevations, which we initially assigned to three taxa: *Calisto grannus grannus* of high elevations in the Cordillera Central (including a specimen representing the *Calisto amazona* phenotype), *Calisto grannus dilemma* (*grannus* individuals with red discal spot on the underside forewing, which includes such taxa as *dilemma*, *micrommata*, *dystacta*, *phoinix*, and *micheneri*) and *Calisto sommeri*, an isolate from Sierra de Bahoruco. The 28 individuals from these nine populations that are identified on the barcode tree as *Calisto grannus grannus*, *Calisto grannus dilemma* and *Calisto sommeri* show geographic, rather than taxonomic, structure. In other words, individuals cluster within populations, separated from other such clusters by 0.5–1.5%, regardless of the taxonomic name applied. For instance, *Calisto sommeri* of Sierra de Bahoruco appears as a sister clade to *Calisto grannus dilemma* from the extreme western portion of Dominican Cordillera Central. The lowland and very common widespread *Calisto obscura* appears to be a sister taxon to the *Calisto grannus* species complex, with a divergence of 5–7%.

5. Within the *Calisto lyceius* species complex (Fig. 4, Clade B), lowland desert isolates such as *Calisto crypta*, *Calisto franciscoi*, and *Calisto lyceius*, despite their superficial morphological similarities, proved to be divergent in their barcodes (ca. 4.5%). *Calisto tasajera* González, Schwartz & Wetherbee, 1991 proved to be their immediate relative, found at the high elevations.

6. A widely-distributed pest of sugar cane, *Calisto pulchella* (Fig. 4, Clade A) showed a high degree of divergence (3.5%) between its two described subspecies (*Calisto pulchella pulchella* from the lowlands and *Calisto pulchella darlingtoni* from the Cordillera Central).

7. Three species endemic to islands other than Hispaniola (*Calisto nubila* Lathy,1899, *Calisto zangis* (Fab., 1775) and *Calisto herophile* Hübner, 1823) appear to be derived lineages of various Hispaniolan taxa (Fig. 3, Clade D and E; Fig. 4, Clade A). Divergence of these island isolates, though high, does not exceed divergence found within the island of Hispaniola.

8. The maximum divergence within the genus (13.3% between *Calisto nubila* and *Calisto grannus*) is almost equivalent to the maximum divergence of *Calisto* from its distant pronophiline relative *Auca* from the southern Andes (14.1%), or from its presumed closest relative *Eretris* (14.4%) (Fig. 4). The average interspecific divergence in *Calisto* was found to be 10%.

## Discussion

As a result of the present “DNA barcode” analysis, it is possible to draw a number of taxonomic conclusions (proposed taxonomic changes are summarized in Table 1). *Calisto grannus* represents a recent and incomplete diversification through allopatric isolation, and for now is best considered as a single species, with *Calisto grannus dilemma*, *Calisto grannus amazona* stat. n., *Calisto grannus micrommata* stat. n., *Calisto grannus dystacta* stat. n., *Calisto grannus phoinix* stat. n., *Calisto grannus sommeri* stat. n., and *Calisto grannus micheneri* stat. n. representing subspecies. Within the *Calisto lyceius* complex, lowland desert isolates such as *Calisto crypta*, *Calisto franciscoi*, and *Calisto lyceius*, despite their superficial morphological similarities, proved to be sufficiently divergent in their barcodes to confirm their species status previously postulated based on male genitalia (Sourakov 2000). The observed divergence within *Calisto pulchella*, which is not only one of the most morphologically divergent species (Sourakov 1996, 1997), but also a widespread and economically important pest of sugar cane (Smyth 1920, Holloway 1933), calls for more research. Interestingly, these results correspond to earlier views (Munroe 1951, Wisor and Schwartz 1985) that there are at least two taxa in *pulchella*, one in the lowlands and another (*Calisto pulchella darlingtoni*) in the Cordillera Central at 3000–4000 ft elevation. Columbus introduced sugar cane to the island around 500 years ago (Deer 1949), so the current distribution of the species is likely different from its historical distribution. Perhaps, pre-Columbus *Calisto pulchella* existed as two non-interbreeding allopatric entities, which continued to maintain no or limited gene exchange following sugar cane introduction, but both were able to adapt a new hostplant. We suggest preserving subspecies status for these two entities until further research can be done, which should include multiple specimens from a number of populations, including studying this butterfly in its wild habitat in association with the native hostplant.

**Figure 5.**
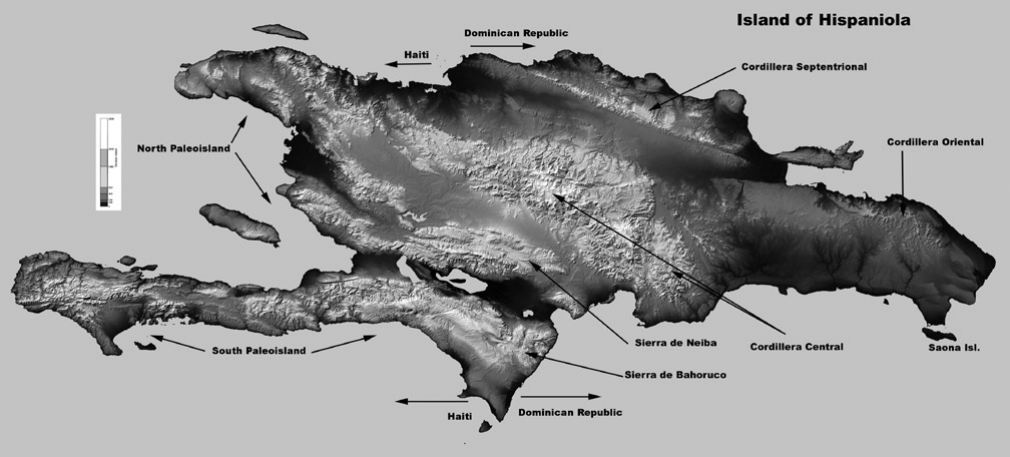
The island of Hispaniola, with some key geological features.

Munroe’s view that *Calisto confusa* and *Calisto debarriera* stat. n. are good species is now supported by our DNA data. Munroe found differences only in aedeagus width/length ratio and immediately cast doubt on his finding: “No fresh material was examined, and such a difference might conceivably be the result of distortion of the preparations.” Munroe examined only four *debarriera* specimens, but stated that “in support of this evidence it may be noted that the material of *debarriera* comes from a limited altitude range, which is entirely contained in both the altitudinal and geographic range of the widely distributed *confusa*.” In other words, Munroe, though only having available a few old collection specimens, already supposed that he was dealing with two sympatric taxa. Future workers reduced *debarriera* to subspecies (e.g., Smith et al. 1984) and even considered it a synonym of *confusa* after their peripatric/sympatric distribution became more and more evident. However, at the same time, additional representatives of *debarriera* were being described as separate species, such as *Calisto neiba* syn. n. and *Calisto gonzalezi* syn. n., based on aberrant isolated populations. Our DNA barcode analysis suggests that *confusa* and *debarriera* are indeed two reproductively isolated species, whose ranges overlap, perhaps as a result of secondary contact following initial speciation through niche partitioning, since *debarriera* is largely a highland species and *confusa* largely a lowland species. A similar confusing situation that existed within the *Calisto hysius* complex, which included *Calisto hysius*, *Calisto batesi* (often listed as *Calisto hysius batesi* (e. g., Munroe 1951)), and *Calisto aleucosticha* stat. n. is now resolved. *Calisto hysius* mostly occurs on the southern paleoisland (Fig. 5) and shows significant divergence from the mostly northern *Calisto batesi*, suggesting that these two are distinct species (Fig. 2). *Calisto aleucosticha*, which was described from a few aberrant females of *Calisto hysius* found on the northern paleoisland by Correa and Schwartz (1986), should be considered a subspecies of *Calisto hysius*.

Non-Hispaniolan island endemics (*Calisto nubila*, *Calisto zangis* and *Calisto herophile*) appear to be derived lineages of various Hispaniolan taxa, indicating several ancient dispersal events from Hispaniola to Puerto Rico, Cuba, and Jamaica. For instance, *Calisto herophile*, which occurs in Cuba and the Bahamas, appears to be a product of dispersal from Hispaniola of the widespread polyphagous *Calisto confusa* or its immediate ancestor. *Calisto nubila*, endemic to Puerto Rico, which bears morphological similarity to the rare and localized Hispaniolan *Calisto eleleus* Bates, 1935 (Fig. 3, Clade D), also most likely have originated by dispersal to Puerto Rico from the Hispaniolan clade. Divergence of these island isolates, though great, does not exceed divergence found within the island of Hispaniola, which suggests that they dispersed from Hispaniola when the genus was already undergoing diversification. The low diversity of species on non-Hispaniolan islands as well as the time-frame of *Calisto* evolution, suggests that such taxa arrived there by accidental dispersal, rather than by land bridges or vicariance as hypothesized previously by Miller and Miller (1989).

*Calisto zangis*, along with *Calisto pulchella* and *Calisto raburni* Gali, 1985, are the most morphologically divergent members of the genus in general wing pattern, male and female genitalic structures, and in the immature stages (at least for *pulchella*, for which life history has been studied) (Sourakov 1996, 1997). DNA barcodes also indicate that these three species are strongly separated, suggesting that the origin of Jamaican *Calisto zangis* is likely an ancient event. The fact that the *Calisto lyceius*/*Calisto tasajera* group of bunch-grass-feeding *Calisto* has a close affinity to cane-feeding *Calisto pulchella* and to the Jamaican *Calisto zangis*, together forming a clade sister to all other *Calisto*, is of great interest. Although the bamboo-feeding *Eretris* were historically regarded as the closest relative to *Calisto* (Miller 1968), our results suggest that the south Andean genus *Auca* may be at least as closely related to *Calisto*, and we suspect that we need to search among lowland bunch-grass feeding satyrines for the closest mainland *Calisto* relative. Feeding on bunch-grasses in low elevation arid habitats may instead be the ancestral state in *Calisto* (e.g., the *Calisto lyceius* complex) that has been retained in other satyrine genera in Central and South America.

Our results highlight the usefulness of DNA-barcode analysis for routine species-level taxonomic work. DNA-barcoding allowed us to confirm previously observed morphological synapomorphies and test theories based on morphology and ecology alone. For example, the fact that the phenotypically divergent species *Calisto archebates* (Ménétriés, 1832), which has a yellow stripe traversing the hindwing underside, appeared as sister species to the *Calisto grannus*/*Calisto confusa*/*Calisto batesi* complex was already hypothesized based on immature stage morphology (Sourakov 1996). Further molecular research involving more genes is necessary to establish a robust phylogeny of *Calisto*.

The evolution of Satyrinae has been linked to the evolution and diversity of grasses (Peña and Wahlberg 2008). The DNA barcode divergence found in this study is associated with apparent ecological niche partitioning by species that inhabit a wide variety of habitats and utilize various host plants. We observe evolution of clades that is associated with shifts to new hostplant groups such as bunch grasses, bamboos, canes, etc. These clade-hostplant associations found today are shown in Figs 2–4. For example, *Calisto arcas* and *Calisto chrysaoros* are two species whose adults are morphologically highly distinctive but whose life histories are poorly known. Sourakov (1996) described the eggs and first instar larvae of these two species and found that while the life history of *Calisto arcas* is surprisingly similar to many other *Calisto*, *Calisto chrysaoros*, which is strongly associated with bamboo, has egg and first instar larva that are highly divergent from the most common *Calisto* phenotype. In the present study, *Calisto arcas* formed a single clade in the middle of BI tree together with *Calisto confusa*/*Calisto neiba* complex (Fig. 3), which supports previously observed morphological synapomorphies. Yet, the average divergence of *Calisto arcas* from other *Calisto* (10–12%) is greater than that of *Calisto chrysaoros* (9–10%).

**Figure 6.**
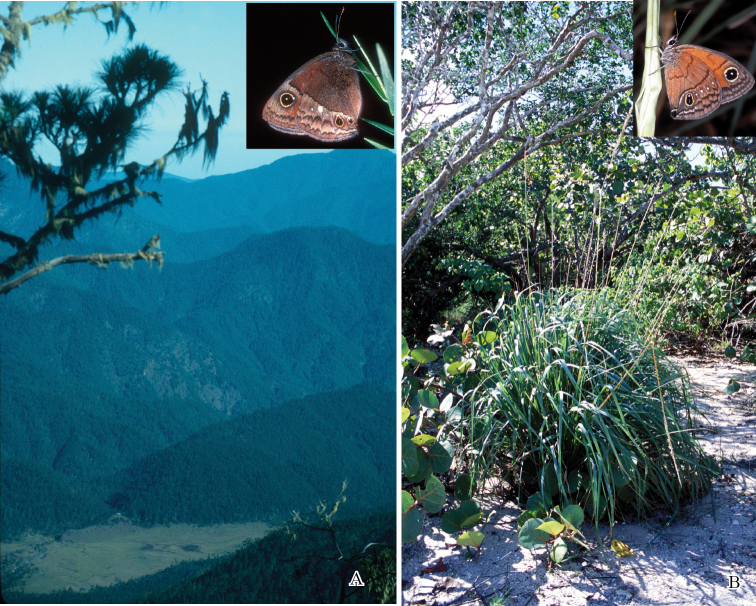
Examples of habitat diversity on the island of Hispaniola. **A** Valle de Bao (1920 m elevation) at the foothill of Pico Duarte (3098 m elevation), covered with bunch grass, *Danthonia domingenis* - a hostplant of *Calisto tasajera* (top right) **B** Arid south eastern coastal habitat in Boca de Yuma, Altagracia provides an environment for sea oats, *Uniola virgata*, and associated *Calisto lyceius* (top right).

The butterfly fauna of Hispaniola has evidently been evolving for many millions of years. For instance, an extinct species of an extant neotropical genus of Riodinidae is known from Dominican fossil amber, dating from 15–25 Myr (Hall et al. 2004). Peña et al. (2011) suggested that *Calisto* might be a remnant of the initial colonization of South America by North American Satyrinae, in which case, *Calisto* might be a very old group. Several authors (e.g., Miller and Miller 1989) have suggested that much of the biological diversification found in *Calisto* may be associated with geological events. Indeed, the geological history of the Greater Antilles, the center of distribution for the genus, is complex. The archipelago originated more than 50 million years ago, and since then the component islands have undergone extensive metamorphosis, with Cuba and Hispaniola separating 20–25 million years ago (Pindell 1994). Though it is tempting to assume some role of geological events in speciation of *Calisto*, it has been shown repeatedly that adaptive radiation process is the main driving force behind evolution of species richness in the Caribbean (e.g., Losos et al. 2006). In our opinion, the genus shows a remarkable degree of diversification in comparison with other Caribbean clades, presumably because of low dispersal ability of these butterflies that interacts with topographic isolation within an island of Hispaniola and with exploitation of different habitats with varying rainfall patterns. Inter-island isolation, of course, also contributed to the overall diversity of the genus. However, it is the incredible diversity of habitats, ranging from the hot, dry deserts of the Hispaniolan lowlands to montane forests and grasslands at over 3000 m in elevation, that is responsible for the todays diversity of *Calisto*. These habitats are so variable due primarily to the high central mountain range, which creates strong gradients of temperature and rainfall. In harsher habitats (e. g., deserts, high mountain tops, peripheral localities (Fig. 6 shows two examples)) where numerous unique adaptations are necessary for survival, species may be very local, not spreading to neighboring areas despite the availability of unlimited resources and seeming absence of interspecific competition.

**Figure 7.**
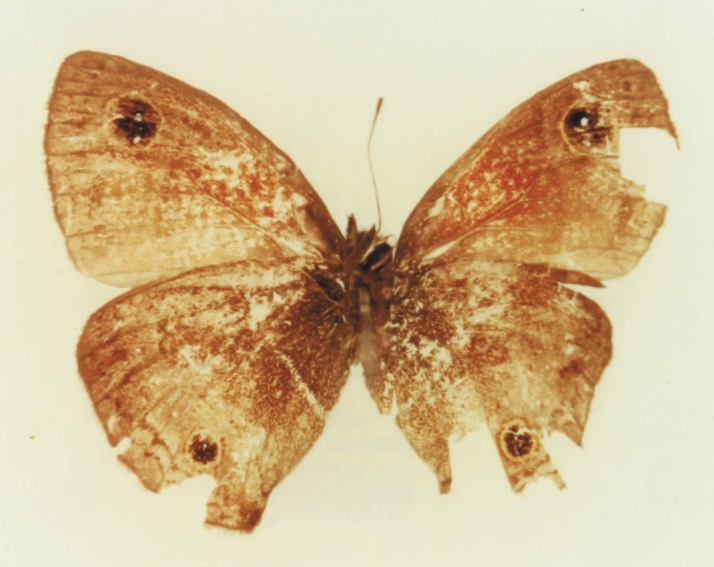
*Calisto montana* holotype (Museum of Comparative Zoology, Harvard, Massachusetts, USA).

Butterflies, especially grass-feeding butterflies in such a hurricane-prone area, have thus had many chances to colonize every possible habitat and island through dispersal. Even though the genus appears more divergent than most other satyrine genera, it does not seem to be old enough to be influenced too much by geological events related to continental movement. Though recognizing the limited ability of a short DNA strand to give precise time estimates for observed divergence, most models assume that 1.5–3.5% divergence roughly equates to one million years of isolation (e. g., Brower 1994, Kandul et al. 2004, Papadopoulou et al. 2010, Vila et al. 2010). Hence, we can hypothesize based on available data that the genus *Calisto* underwent continuous diversification for some 4–8 Myrs, and thus ancient geological events of continental movement are unlikely to be a factor. Instead, it seems most likely that the diversification of *Calisto* into these numerous different habitats represents traditional Darwinian adaptive radiation, as suspected for other groups of Caribbean insects and vertebrates (e.g., Losos and Schluter 2000; Woods 1989; Liebherr 1988).
